# Integration and Testing of a High-Torque Servo-Driven Joint and Its Electronic Controller with Application in a Prototype Upper Limb Exoskeleton

**DOI:** 10.3390/s21227720

**Published:** 2021-11-20

**Authors:** Manuel Andrés Vélez-Guerrero, Mauro Callejas-Cuervo, Stefano Mazzoleni

**Affiliations:** 1 Software Research Group, School of Computer Science, Universidad Pedagógica y Tecnológica de Colombia, Tunja 150002, Colombia; mauro.callejas@uptc.edu.co; 2 Department of Electrical and Information Engineering, Polytechnic University of Bari, 70126 Bari, Italy; stefano.mazzoleni@poliba.it

**Keywords:** robotics, servomotor, joint, actuator, exoskeleton, mechatronic systems, control, upper limbs, modeling robotic systems

## Abstract

Mechatronic systems that allow motorized activation in robotic exoskeletons have evolved according to their specific applications and the characteristics of the actuation system, including parameters such as size, mechanical properties, efficiency, and power draw. Additionally, different control strategies and methods could be implemented in various electronic devices to improve the performance and usability of these devices, which is desirable in any application. This paper proposes the integration and testing of a high-torque, servo-driven joint and its electronic controller, exposing its use in a robotic exoskeleton prototype as a case study. Following a brief background review, the development and implementation of the proposal are presented, allowing the control of the servo-driven joint in terms of torque, rotational velocity, and position through a straightforward, closed-loop control architecture. Additionally, the stability and performance of the servo-driven joint were assessed with and without load. In conclusion and based on the obtained results, the servo-driven joint and its control system demonstrate consistent performance under the proposed test protocol (max values: angular velocity 97 °/s, torque 33 Nm, positioning RMSE 1.46°), enabling this approach for use in various applications related to robotic exoskeletons, including human performance enhancement, rehabilitation, or support for daily living activities.

## 1. Introduction

The current technological and industrial revolution, and the appearance of various manufacturing techniques, promote progress in the design and development of wearable robotic exoskeletons attached to different limbs of the human body [1]. Currently, robotic exoskeletons (also called exosuits) are one of the most versatile device families that have been successfully developed [2,3]. A broad range of applications has made robotic exoskeletons a key research topic [4], impacting cross-cutting areas beyond engineering [5]. Exoskeletons have become highly relevant, from systems with industrial applicability [6], through the enhancement of human capabilities [7], to complex rehabilitation systems for both upper and lower limbs [8,9].

Although developments often focus on providing mechanical support, other factors such as implementation cost, power consumption, or device weight take a back seat in wearable or portable systems [10]. This could limit the practical use of the proposed actuators in applications where these improvements are required [11].

However, with the recent advancement in mechatronic systems as well as embedded control techniques, new milestones in design, size, weight, and capabilities have been reached [12]. Regardless of their application, the development of actuation systems is essential for the improvement of various mechatronic systems that continue to be updated [13] as it allows for increased efficiency in mechanical and electrical terms.

From an applied control engineering perspective, control system development and enhancement have allowed increasing applications of existing actuation systems [14,15], making hardware and software co-design an essential process. In addition, the use of advanced processing and control techniques allows for improved actuator stability and response times. Consequently, more demanding applications can arise from the implementation of these advantages [16].

Considering the above, this document proposes the integration of a high-torque, servo-driven joint and its electronic controller, taking as a study case the actuation of an upper limb exoskeleton prototype. Additionally, the paper presents the test results conducted to determine the stability of the actuation system in different scenarios, including the technological integration, angular velocity maximums, torque output, and positioning error. The objective of these analyses is to determine the preliminary performance of the proposed joint under loaded and unloaded motion conditions.

As shown in the Discussion section, the main contribution of this work is the development of a single structure containing a high-torque actuator and its control system, proposing a robust embedded system with balanced power consumption and demonstrating low positioning error with speeds considered high and suitable for various applications.

This paper is sequentially organized in different sections, as mentioned below. Section 2 presents a brief contextualization based on background research related to the development of mechatronic actuation systems in robotic exoskeletons. Section 3 describes the central proposal, emphasizing the materials and methods used for its development, including the used protocol for the servo-driven joint testing. Section 4 shows the implementation of the proposal and compiles the achieved results. Section 5 present the discussions and comparison of the findings with the previously reviewed literature. Finally, Section 6 offers conclusions and possibilities for future work derived from this research.

## 2. Related Works

This section provides a brief compilation of relevant studies that encompassed mechanical actuation systems in robotic exoskeletons, including other surrounding topics such as embedded electronics and specific control algorithms. The reviewed papers provide background information on the key elements addressed in this research. The scope of this study is limited to those systems with servomotor-based actuators, which serve as a direct reference point for the comparison and subsequent discussion of the results obtained in this paper.

### 2.1. Servo-Driven Systems in Robotic Exoskeletons

As a starting point from the mechanical side, the gearbox designs presented in [17] are relevant when exploring different ratio configurations and their potential applications. This review highlights the inclusion of several compact mechanical transmission systems, which can serve as a benchmark in the design of more compact, lightweight, and functional transmissions.

As robotic systems continue to be explored, and regardless of the final application of the exoskeleton, servomotor-based drive systems can have different implementations. One of the most comprehensive papers shows the development of a modular and compact drive system for a lower limb exoskeleton [18]. It highlights the high response speed of the proposed control system, reaching a maximum of 110 °/s (2.0 rad/s peak).

The research conducted in [19] shows a compact structure offering high torque (absolute maximum values of approximately 30 Nm) in knee-ankle joint motion assistance applications. This study shows that the maximum speed is up to 180 °/s (30 RPM). In addition, various tests are performed on the actuator, which also conclude that it has sufficient torque for lower-limb-rehabilitation applications, although it is restricted in software to avoid thermal overstrain.

In the same sense, [20] uses a series of servo-actuators for the development of a walking assistance robot. Although their application focuses on the development of Body Weight Support (BWS) systems as in [21], these systems are considered a suitable approach to the use of servo-actuators for demanding applications in terms of torque and versatility.

Some other developments show devices implemented to allow the mobility of the upper limbs. Such is the case of the system proposed in [22], which provides high speed (reaching a maximum value of 0.3 m/s) combined with precise position control and moderate torque for rehabilitation purposes. Furthermore, and derived from the presented data, it is clear that the effective range of motion of the exoskeleton is wide, reaching reference values for elbow flexion-extension of up to 140°.

Finally, in [23], an experimental design of an arm motion assistance device is presented. This device is characterized by its high portability and modularity, as well as its simple design, which allows rapid prototyping. The results establish an estimated total torque of 5 Nm, an effective range of 48.62°, and an average current drawing of 0.3 A.

### 2.2. Electronic Systems and Control Techniques

In terms of electronic devices and strategies for controlling servo-driven systems, there are several remarkable approaches. The work developed in [24] is particularly significant since it reviews several advanced control strategies for servo devices, mainly using sliding methods. These advanced control methods enable better trajectory tracking as well as active disturbance rejection, which can improve actuator performance in applications that require higher precision in motion execution. Another controller based on sliding modes excels in performance with servo actuators [25], achieving signal establishment and tracking in less than 20 s and a maximum positioning error of 0.4°. These results are exceeded in [26], where a similar controller achieves stable position establishment in less than 12 s and an average error of 0.21°.

The research of [27] stands out, in which an integrated cascaded-controller system was designed to complete rehabilitation training sessions. It has near-immediate response rates on an electrically actuated joint, although no readily derivable numerical results are provided compared to other proposals. The work highlights that by combining the computational capability of a DSP and the processor power, cascade controllers can adapt different load perturbations and make the active forces of the subjects reach maxima.

The device previously reviewed in [23] has a control system built with Arduino and other easily accessible elements, similar to the proposal shown in [18]. Although the system responsiveness is good, as in the case of [28], the integration of different signals types adds value to the proposed control system. The latter work specifically highlights a response time in the order of milliseconds, with an amount of error ranging from 7% to 9% (20°–23°) under load conditions.

Regarding the field of upper limb robotic actuators, the results shown in [29] are significant, as they use digital filtering techniques to improve system performance. Control signal stabilization is achieved in less than 1 s, reducing the actuator positioning error close to 20%.

The research developed in [30] provides excellent results in terms of positioning error reduction, reaching values close to zero, ensuring the final uniform delimitation of the lower limb exoskeleton robot. The technique employed for trajectory tracking was based on Udwadia-Kalaba theory, which constitutes a novel, robust and adaptive control system. Although the results are based on simulations, experiments are expected to match the paper with high fidelity.

Finally, as for control strategies based on position and force in a single unit, the work presented in [31] is characterized by achieving low absolute error, although its application is based on linear actuators. The method is suitable for both trajectory tracking during free motion and interaction force control during contact between a controlled mechanical system and its environment. The proposed algorithm is simple and easy to implement, allowing its deployment in embedded systems added to conventional actuators, taking a step towards “smart actuators”.

## 3. Materials and Methods

This section presents the collection of materials and methods used for the development of the servo-driven joint. It includes the description of the actuation system; the upper limb robotic exoskeleton (based on [32]), where the proof of concept is performed; the basic control system that drives the joint; and finally, the test protocol designed to determine the performance of the integrated systems.

### 3.1. Servo-Driven Joint and Exoskeleton Prototype

**Figure 1:**
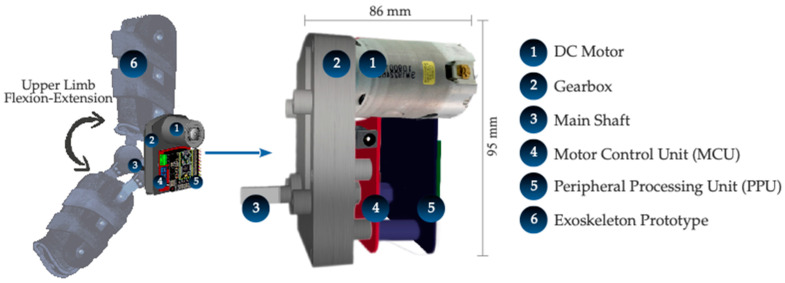
Actuation system based on a DC motor, a gearbox, and electronics, attached to an upper limb exoskeleton prototype for flexion-extension movement.

The servo-driven joint is composed of a high-speed DC motor coupled to a mechanical transmission that provides an optimal ratio of high torque and moderate speed for the intended application. As seen in Figure 1, the motor frame and gearbox are coupled to a rotational hinge that binds two segments of the prototype upper limb exoskeleton together, providing actuation at the elbow joint level for 1-DoF extension and flexion motion. On the other hand, the specifications of the DC motor and the gearbox used are presented in Table 1 and Table 2, respectively.

The components selection, in particular the 560:1 transmission ratio, allows obtaining a theoretical maximum rotational speed of 20 RPM (120 °/s) and a maximum theoretical torque around 41 Nm at the output of the servo-driven joint.

### 3.2. Control System

To make the best use of the available space and create a compact system, two circuit boards are mounted on the mechanical system described above to allow the implementation of the control system. The control system consists of the Motor Control Unit (MCU) and the Peripheral Processing Unit (PPU), with redundant (hardware-hardened) microcontroller-based units.

**Figure 2:**
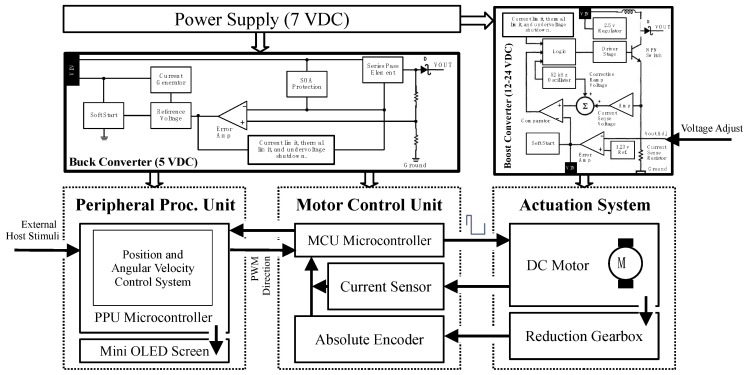
Functional block diagram integrating the power converters, control units, sensors, and the actuation system.

In addition, the circuit board comprising the MCU also contains sensors that continuously monitor the behavior of the actuation system. (1) A current sensor is included, allowing a further algorithmic torque calculation. (2) An embedded high-precision absolute encoder determines the position of the main shaft at the gearbox output and before coupling to the exoskeleton, eliminating possible structure oscillations. This encoder type is less susceptible to magnetic disturbances, thus increasing its reliability. Figure 2 below shows the functional block diagram of the proposed system.

**Figure 3:**
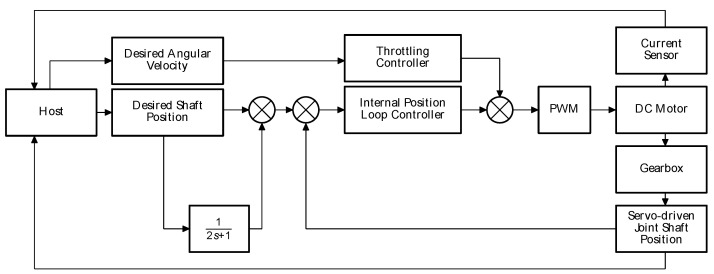
Diagram of the simple closed control loop proposed for this development.

As for the controller architecture, a classical strategy based on a PID-type controller is used. This controller is designed as a closed-loop system, whereby the position of the main axis of the joint actuator (measured by the absolute encoder) is fed back into the system. Additionally, other variables (such as those produced by the current sensor) are used to calculate the torque exerted (although no direct control over this parameter is realized). The desired angular velocity is also taken into account, thus controlling a PWM modulator that regulates the direct speed of the DC motor, directly impacting the speed of the final axis of the joint. A diagram of the simple controller implemented in this development is shown below in Figure 3.

This controller was designed to create a simple loop with a hardware-effective structure. The control methodology is based on obtaining an initial reference value from the host computer, both in terms of the desired position and the desired angular velocity. The desired position is filtered and transferred to the controller, which manages the position of the DC motor using a PWM modulator. The PWM modulator also takes the desired angular velocity as a reference to move the motor at different speeds. It should be noted that the throttle-handling feature is constituted by an external static controller that applies a modification in the time domain of the angular velocity reference.

When this is transferred to the final actuator (actuated join main shaft) through the gearbox, the absolute encoder measures the position and feeds the signal back to the controller, which dynamically makes the necessary adjustments to maintain the desired reference value. Independent of this closed control loop, the current sensor sends the information directly to the computer to provide data about the current draw of the motor during the execution of the motion. Although this controller does not directly manage the torque at the actuator output, an additional closed-loop stage using the current drawn by the motor can be included to allow this functionality.

Characteristically, the frequency of feedback of information to the control loop is 120 Hz. Likewise, the position update frequency from the host (when tracking a trajectory, for example) is 30 Hz. This allows the controller to quickly adapt to externally produced signal changes and properly achieve the final axis position setting. The characteristic latency of the controller was not determined.

### 3.3. Testing Protocol

As specified in Table 3, a testing protocol is proposed to determine the correct functioning and performance of the servo-driven joint. This test protocol is independent of the chosen application, as it allows establishing some of the mechanical and electrical characteristics of the actuator together with the proposed embedded controller.

## 4. Results

This section shows the results obtained following the test protocol established in Table 3. It is highlighted that the prototype exoskeleton coupled to the servo-driven joint was used on a healthy human subject for load testing after performing the non-loaded tests, verifying the safety measures framed within the research project.

### 4.1. Functional Integration

**Figure 4:**
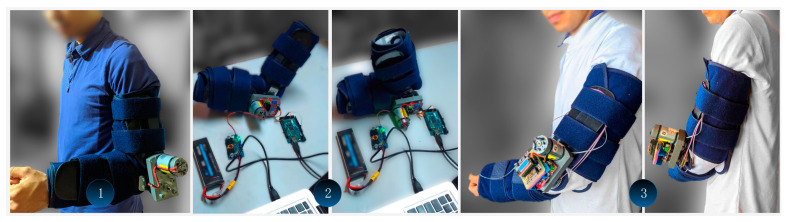
Servo-driven joint, exoskeleton prototype, and control system coupling.

To test the functionality of the servo-driven joint and exoskeleton prototype, as well as its basic functionality, a functional integration must be performed. Figure 4 shows the integration of the different components of the system according to the defined methodology. This integration process takes place in three stages. In stage 1, the motor and gearbox are coupled and fitted into the exoskeleton prototype. In stage 2, the control elements (MCU and PPU) are incorporated into the system as partially integrated elements while initial validations are performed. Finally, in stage 3, a single integrated drive and control system is assembled.

The activation and operation commands were sent through a computer to the PPU via a USB connection. The same connection was used to collect the MCU parameters in real-time. There is an additional safety mechanism that allows the immediate disconnection of the power supply of the prototype using a switch. The electrical power supply is noticeable on stage 2, where a high-density LiPo battery (7.4 V, 5300 mAh, Current Rating 30 C) is connected to the buck-boost conversion system.

The integration of the functional elements and their operability is successfully tested, as motion transfer is performed from the servo-driven joint to the exoskeleton system in both loaded and unloaded scenarios. The maximum motion range in the servo-driven joint is found from 0° (maximum extension) to 300° (maximum flexion). This range must be algorithmically limited as it exceeds the mechanical properties of the exoskeleton prototype. The overall amplitude is set from 0° to 145° at maximum.

### 4.2. Angular Velocity

For the experimental measurement of the maximum servo-driven joint angular velocity, a 24 VDC nominal voltage is set on the boost converter. Theoretically, the maximum velocity with this configuration is 120 °/s using the gearbox gear ratio (560:1) as the calculation basis. The implemented controller in the MCU has a throttle curve defined by a sigmoid function for both flexion and extension, which smoothes the motor starting and stopping procedures, thus avoiding current surges. The throttle function is defined as shown in Equation (1).

(1) $$f\left(x\right)=1\cdot {\left(1+e^{-c_{1}*\left(x-c_{2}\right)}\right)}^{-1}$$

**Figure 5:**
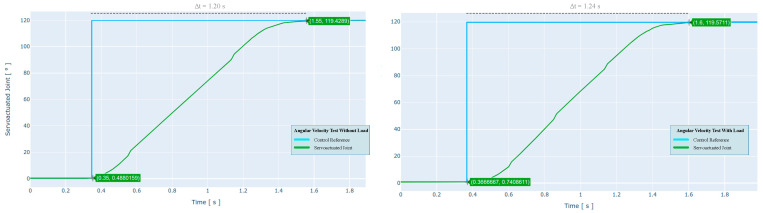
Characteristic response curves over a 0° to 120° movement using a 24 VDC nominal voltage at the input of the actuation system. The figure shows the test without and with load, respectively.

The DC motor is activated when the control signal is sent from the host computer to the PPU and transmitted to the MCU, where the throttle function is applied. For this test, a step signal is sent, which sets the set point at 120°. Therefore, the trajectory starts at 0° (maximum extension) until it reaches 120° (flexion). The resulting experimental response curve is shown in Figure 5, which is used to analytically determine the actuator velocity as a function of system response time.

During non-load activation, the servo-driven joint travels 120° in 1.20 s. This leads to a maximum experimental angular velocity of 100 °/s (1.74 rad/s, 16.67 RPM), corresponding to 83.3% of the previously calculated theoretical velocity. Concerning the motion with load, the servo-driven joint travels 120° in 1.24 s. This leads to a maximum experimental angular velocity of 97 °/s (1.69 rad/s, 16.17 RPM), equivalent to 80.6% of the theoretical velocity calculated above.

### 4.3. Torque

**Figure 6:**
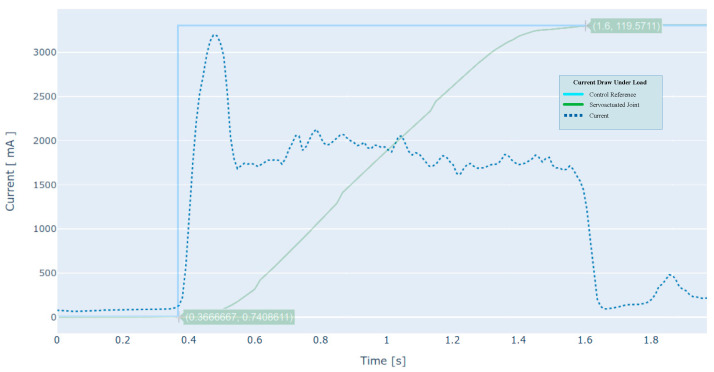
Typical current draw produced by the motor during the motion under load condition.

For the analytical torque calculation, the current draw is measured during the above test protocol. This information is graphically represented in Figure 6.

Since the current is not a constant value over time and fluctuates according to load conditions and acceleration, among others, a summary of the average torque values is presented in Table 4 below.

As long as the servo-driven joint reference does not change (standby), there is a slight current draw due to continuous end-position adjustments by the controller, producing soft movements in the DC motor. Regarding the current sensor used for these measurements, it has an operating measurement range of ±5 A with a typical sensitivity of 185 mV/A, a typical impulse response time of 5 μs, and a bandwidth of 80 kHz.

The torque is determined analytically according to Equation (2), where V is the motor voltage, I is the measured current in Amperes, N is the measured RPM, and E is the efficiency of the gearbox.

(2) $$T=E\cdot \left(V\cdot I\right)\cdot {\left(\frac{2\cdot \pi \cdot N}{60}\right)}^{-1}\;\left[\mathrm{Nm}\right]$$

Taking this into account, Table 5 presents the result of the analytically found torque according to the current measurements.

The analytically found torque is lower than the theoretical torque determined with the conversion ratio, possibly due to the efficiency of the gearbox during mechanical conversion. However, the values obtained theoretically are consistent with the measurements performed, establishing a suitable confidence range.

### 4.4. Positioning

The positioning of the servo-driven joint is performed by the integrated electronics. While the system automatically follows a control reference, control signals (PWM) are automatically generated using closed-loop feedback information. The error is calculated using Equation (3): *F_m_* is the flexion position measured at the encoder, *E_m_* is the extension position measured at the encoder, and *F_r_* and *E_r_* are the reference flexion and extension positions as set by the control signal.

(3) $$E_{\mathrm{abs}}=\left(E_{m}-E_{r}\right)+\left(F_{r}-F_{m}\right)\;\left[{}^{\circ}\right]$$

**Figure 7:**
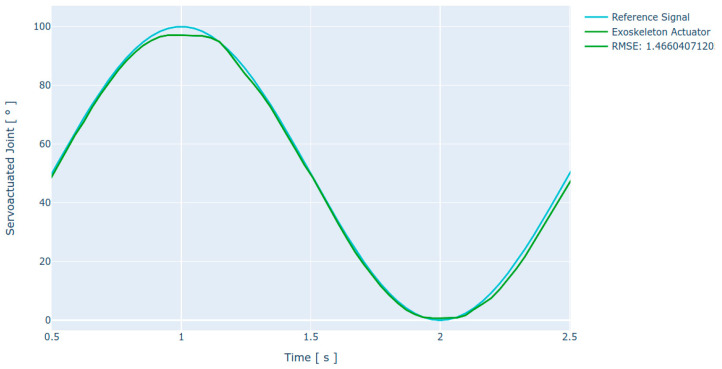
Characteristic response curves over a 0° to 120° movement using a 24 VDC nominal voltage at the input of the actuation system. The figure shows the test under load conditions.

The absolute error for the servo-driven joint motion was 1.06° (without load) and 1.17° (with load). This error is low and is intended to be compensated by the control system. However, it is necessary to calculate the RMSE over the joint motion over a pattern, giving a more realistic insight into the system’s behavior when used in real-life applications. Tracking of a sinusoidal reference pattern is satisfactory, resulting in an RMSE of 1.46°. It is observed that the motion is accurate at a high angular velocity, reaching 96 °/s, which coincides with the results obtained with the step stimulus. The response curve obtained is presented in Figure 7.

### 4.5. Stability

**Figure 8:**
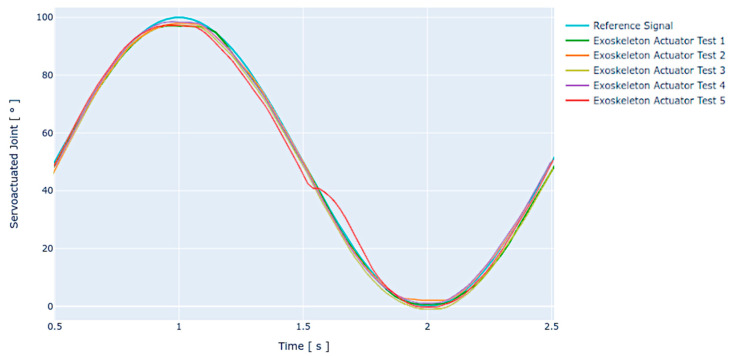
Stability test over a 0° to 120° movement using a 24 VDC nominal voltage at the input of the actuation system. The figure shows the tests under load conditions.

An additional test aimed at determining the stability of the joint actuator was performed using the positioning test protocol as a basis. For the system stability test, the routine was performed concurrently in five cycles with the parameters described above (joint amplitude 120°, nominal input voltage 24 VDC, and expected angular velocity 96 °/s). The test results are graphically shown in Figure 8 below.

Following a qualitative analysis of the obtained results, the system presents expected behavior with minor variations between executions. As for the angular velocity, the system presents minimum lags or leadings that are compensated by the proposed controller within the expected response limits. These variations in angular velocity may be due to changes in the voltage flow through the controllers or to the system dynamics itself.

Regarding the positioning behavior, a pitfall occurs via signal flattening, failing to reach the maximum value of the peak of the reference sine wave. This problem is concurrent across all tests, presenting a similar flattening in magnitude each time. In the same way, smaller deviations occur, reaching the valley of the sinusoidal reference signal, whereas the position of the joint actuator seems to exceed the established limit in some cases. Considering this behavior, it is believed possible to adjust the controller in a future revision to compensate for the offset of the actuator response.

In the last repetition of the stability test (Exoskeleton Actuator Test 5), an anomaly occurs in the controller response, leading to a partial loss of reference signal tracking, which constitutes a special case. Although this anomaly is subsequently compensated by the controller, the settling time of this correction was greater than 300 ms, which is considered a slower response time than the average in other position correction scenarios. However, it should be noted that the tracking of the reference waveform was satisfactory after the required recovery time.

Table 6 shows a summary of the RMSE obtained for each of the tests performed in this protocol.

## 5. Discussion

The proposed system has some remarkable features compared to other reviewed studies. Regarding the effective joint amplitude range, the proposed system achieves a maximum flexion of 140°, which is 5° larger when compared to previous works such as [22]. Additionally, the total amplitude of this proposal is 96° wider compared to [23]. Considering that the natural range of elbow extension is 2° ± 9°, and elbow flexion is 142° ± 12° [33], the proposed device covers the typical upper limb working ranges in various applications such as human limb enhancement or rehabilitation using active devices.

As for the angular velocities achieved with the proposed servo-driven joint, the joint is found to have a satisfactory velocity range that meets the above tasks, effectively reaching 97 °/s when tested under load. It has been shown that the proposed servo-driven joint has a longer settling time, reducing its maximum and average speed. Some of the previously reviewed works are capable of higher final velocities, which can be improved in this proposal. In this section, some results stand out, such as those in [18] (110 °/s peak), in [19] (180 °/s peak), and in [22], although in the latter, the final angular velocity reached (expressed as linear velocity around 0.3 m/s peak) is not certain.

In terms of the nominal torque under loaded tests, the proposed servo-driven joint can develop a maximum torque of 34 Nm, exceeding the nominal torque of other previously reviewed works, such as [19] (30 Nm limited due to thermal control under the actuator) and [23] (reaching 5 Nm, although this is sufficient for the application of the system).

Regarding the trajectory executed by the joint, it can be observed that the acceleration curve allows softening the movement in the exoskeleton. The curve facilitates the reduction of the load on the voltage converters and creates a smooth movement according to the arm biomechanics. This can be considered an advantage when performing precise tasks. This result is better in terms of smoothness and noise reduction compared to other works such as [23,28,29].

It is noted that the simple controller proposed for this servo-driven joint has a characteristic response time in the order of milliseconds, comparable to the results obtained in [27,29,31]. The obtained results in this proposal surpass those evidenced in [25] (with a settling time close to 20 s maximum) and in [26] (with a settling time close to 12 s maximum).

As for the absolute accuracy of actuator movements, controllers using sliding modes and other techniques evidenced in the review allow a significant error reduction. This proposal yields an absolute error of 1.17°, which is a larger error value when it is compared to the results shown in [25] (0.4° absolute error), and compared with the results shown in [26] (0.2° average error). However, this proposal is better compared to [28,31], since the latter has a higher RMSE error (in some cases exceeding 20°). Despite this, this proposal can be improved in terms of the absolute accuracy of the servo-driven joints and achieve better reference values, as shown in [30].

Finally, some tests show that the proposed controller for this servo-driven joint performs faster and without overshooting when it comes to tracking a static or dynamic reference, compared to works such as [22,29], where the tracking of the proposed trajectories is slightly better. These works do not offer a standardized measure of error, so quantification of the improvement is not possible in arithmetic terms. Similar comparisons were made in [31], where although adequate trajectory tracking is performed, flattening occurs, which impacts the final accuracy of the system.

**Figure 9:**
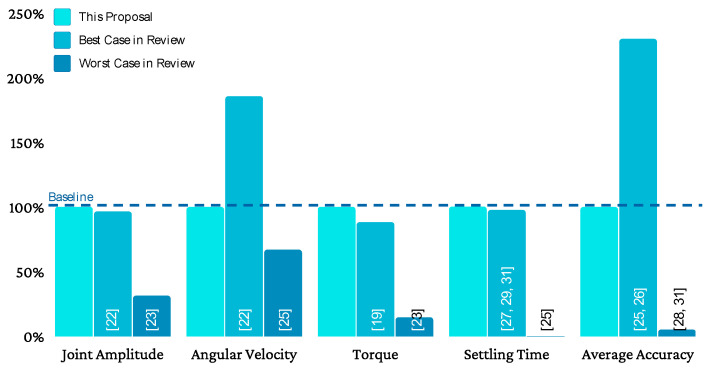
Graphical summary of the results obtained contrasting the best and worst results documented in the state of the art for each comparison item.

Figure 9 shows a graphical summary of the results obtained in this proposal compared to some of the works shown in the state of the art, specifically contrasting the best and worst documented results for each comparison item. For reading unification, regardless of the measurement unit, the results are expressed as a percentage. Those results that are better than those obtained in this proposal are above 100%, while results that are worse than those obtained in this proposal are below 100%.

## 6. Conclusions and Future Work

This paper described the integration and testing of a high-torque, servo-driven joint and its embedded electronic controller with application in a prototype upper limb exoskeleton. By designing the servo-driven joint with a high-ratio transmission gearbox, a high torque output with a peak of 34.3 Nm under load is achieved, which enables the movement of the mechanical structure and the human arm in different tasks that can range from industrial, human augmentation, and rehabilitation.

The angular velocity of the proposed servo-driven joint reaches a peak value of 97 °/s under load. Although this speed is an average value and is limited by the control system to avoid current surges in the boost converter, it is adequate for the applications mentioned above.

Lastly, low absolute error (1.17°) and root-mean-square error (1.46°) values are obtained, which implies that the developed direct control strategy has suitable characteristics for the precise motion of the joint. Additional features include the low weight of the joint (approx. 590 g while quantifying the electronic system), allowing the application of this system in wearable prototypes. Additionally, noteworthy is the functional integration of all elements, with an embedded control system that is capable of performing the tasks without further bulk within the actuator design itself.

As future work, the use of better control algorithms (such as those presented in [24,25,26]) is proposed to decrease the positioning error. The use of other throttling functions can be explored, aimed at avoiding current surges in the power converters but improving the speed response of the servo-driven joint. Additionally, decreasing the weight and size of the proposed joint and its electronic controller is important to gain versatility in the use of the system, thus enabling possible applications in robots integrated with the human body.

It should be noted that beyond the design of the actuation system and its electronic controller, future work also includes the improvement of the specific application described in this work (robotic exoskeleton prototype). It is necessary to consider critical aspects such as cooperation with the movement of human limbs and their protection against fatigue or overload. This requires the use of a larger number of sensors and new control strategies that use the principles shown in this work and that allow the refinement of a more concrete application based on this type of integrated actuator.

## Figures and Tables

**Table 1:** DC motor specifications.

Motor Parameter	Value
Motor Type	DC Brushed
Rotational Speed	11,200 RPM
Torque	75 mNm
Nominal Voltage	12–24 VDC
Nominal Current	4500 mA
Weight	285 g
Material	Steel

**Table 2:** Gearbox specifications.

Reducer Gearbox Parameter	Value
Gearbox Type	Compound Spur Gear
Max. Input Rotational Speed	12,000 RPM
Ratio	560:1
Efficiency	0.79
Output Shaft Diameter	8 mm
Weight	278 g
Material	Steel

**Table 3:** Test protocols of the servo-driven joint and its control system.

Item	Test Protocol
Functional integration	Motion transfer checks from the servo-driven joint to the exoskeleton mechanism and its maximum flexion-extension range.
Angular velocity	Experimental determination of the maximum angular velocity at a 24 VDC nominal voltage, both with load and without load.
Torque	Analytical calculation of the maximum delivered torque. The current draw value is recorded during the angular velocity test.
Positioning	Analytical and experimental determination of the positioning error at the maximum registered angular velocity.

**Table 4:** Current draw measurement during the previous test protocol.

Measurement	Registered Value without Load	Registered Value with Load
Peak Current (absolute)	1780 mA @ 16.67 RPM	3061 mA @ 16.17 RPM
On-duty Current (avg.)	1425 mA @ 16.67 RPM	2962 mA @ 16.17 RPM
Standby Current (avg.)	392 mA	430 mA

**Table 5:** Torque calculation based on the recorded current consumption.

Measurement	Analytical Value without Load	Registered Value with Load
Peak Torque (absolute)	19.3 Nm	34.3 Nm
On-duty Torque (avg.)	15.5 Nm	33.2 Nm

**Table 6:** Summary of the RMSE obtained for each of the tests performed in the stability test protocol.

Test	RMSE
Exoskeleton Actuator Test 1	1.46°
Exoskeleton Actuator Test 2	1.58°
Exoskeleton Actuator Test 3	1.13°
Exoskeleton Actuator Test 4	1.35°
Exoskeleton Actuator Test 5	2.32°

## Data Availability

Not applicable.
